# Cutaneous Squamous Cell Carcinoma of the Head and Neck: A Retrospective Analysis of Loco-Regional Recurrences and Survival Rates Over a Consecutive 10-Year Period

**DOI:** 10.7759/cureus.64805

**Published:** 2024-07-18

**Authors:** Omar Yaqoob, Marco Dalle Carbonare, Deepak Komath

**Affiliations:** 1 Oral and Maxillofacial Surgery, Royal Free Hospital, Royal Free London NHS Foundation Trust, London, GBR

**Keywords:** risk factors, recurrence, survival rates, cutaneous, head and neck, scc, skin

## Abstract

Introduction: Cutaneous squamous cell carcinoma (cSCC) is one of the most common skin cancers worldwide. Due to the ever-increasing sun exposure and life expectancy, cSCCs are increasing worldwide. The aim of our study was to identify specific risk factors leading to local and regional recurrences, determine patients’ survival rates, and identify best practices for the management of cSCC.

Methodology: This study retrospectively analyzed 1197 head and neck cSCCs in 945 patients who consecutively presented to the clinics from January 2007 to December 2016. Patients were followed up for a minimum of 18 months.

Results: A total of 29 patients (3%) developed loco-regional recurrences (26 local, one regional, and two both local and regional) with a median time to recurrence of 25 (range, 1-81) months. The mean follow-up was 32 (range, 5-90) months. Treatment modality (p=0.027), depth of invasion (p<0.001), diameter > 20 mm (p<0.001), gender (p=0.022), histological differentiation (p<0.001), site of the lesion (p<0.001), perineural and intravascular invasion (p<0.001), positive lymphadenopathy (p=0.021), immunosuppression (p<0.001), and history of treatment (p=0.008) proved to be strong predictors for loco-regional recurrences. At one and five years after diagnosis, 95.6% and 59.9% of all patients were recurrence-free, respectively. The median survival time from recurrence was 2.6 years.

Conclusion: Our study identifies prognostic indicators for reoccurrence by analyzing data from a large continuous cohort in the management of cSCCs.

## Introduction

Cutaneous squamous cell carcinoma (cSCC) is the second most common non-melanoma skin cancer after basal cell carcinoma (BCC) among fair-skinned individuals, with an estimated lifetime risk of 7-11% [1]. The incidence of these tumours is increasing due to excessive exposure to ultraviolet radiation and increasing life expectancy [2-6]. One-fifth of non-melanoma skin cancers are cSCCs, with 50-75% occurring in the head and neck region [1,2]. Despite being less common than BCCs, cSCCs bring a potential for metastatic spread and therefore cause increased morbidity and mortality. Currently, while factors affecting the metastatic potential of cSCC exist, guidance specifically looking at the head and neck region only is sparse [7]. With an early-stage diagnosis, a large proportion of these tumours in the head and neck region are curable due to their predominantly localised growth pattern. It is estimated that recurrence rates and lymphatic spread are normally below 10% and 5%, respectively [1,3,4,8-10].

In previous cohort studies, factors such as tumour diameter > 2 cm, depth of invasion > 2 mm, perineural invasion, poorly differentiated subtype, and ear and lip as primary affected sites have been advocated as predictors for recurrence and poor survival rate [2,3,8]. The American Joint Commission on Cancer (AJCC) have considered these features to determine the T stage, where ≥ 2 cm in maximum diameter qualifies a tumour as T2 [11]. Further features such as positive lymphadenopathy, neuropathic symptoms, immunosuppression, growth rate, proximity to the parotid region, history of treatment, and treatment modality have been used as predictors of high-risk cSCC [5,10,12-17]. 

Given the lack of uniformity in the literature on risk factors that predict loco-regional recurrences, the aim of our study was to establish the demographic features in patients with cSCC, evaluate features that can be identified as reliable predictors for recurrent cSCC, calculate the recurrence and survival rates, and identify the best treatment modality for cSCCs of the head and neck region.

## Materials and methods

This was a retrospective cohort study conducted at the Barnet Hospital, Royal Free London NHS Foundation Trust, London, United Kingdom. All data collected was obtained in accordance with the Trust guidelines and anonymised. Patients with cSCCs were identified from each MDT meeting outcome sheet between January 1, 2007, and December 31, 2016, and selected for inclusion in the study.

The inclusion criteria were: (i) Patients diagnosed with cSCC in the Head and Neck region and discussed at the MDT meeting between January 1, 2007, and December 31, 2016, (ii) Patients treated with curative intent, (iii) Patients who underwent Mohs micrographic surgery (MMS), wide local excision (WLE) with or without adjuvant radiotherapy, therapeutic curettage and cautery (C+C), and radiotherapy as treatment modalities.

The exclusion criteria were: (i) Patients who received palliative treatment, (ii) Patients who sought private treatment, (iii) Keratoacanthomas diagnoses, (iv) cSCC in situ, (v) Patients who did not maintain regular follow-up for at least 18 months, (vi) Patients with incomplete records.

Data collection occurred on the following parameters: age, sex, site of lesion, treatment modality, presence of positive margins, tumour diameter, depth of invasion, histological differentiation, perineural and intravascular invasion, presence of lymphadenopathy, immunosuppression, and date and location of recurrences. The depth of invasion was divided into three sub-groups (< 2 mm, 2-4 mm, and > 4 mm). Tumour size was grouped into two categories (< 2 cm and > 2 cm). Histology was classified as well into moderately or poorly differentiated subtypes.

Statistical analysis 

Patients’ characteristics and lesions were summarised as frequency (percentage). Trends in the use of WLE and recurrence rates were assessed using the Chi-square test. Rates were assessed with Fisher’s exact test. The time period from diagnosis to identification of first recurrence was termed a recurrence-free period. Kaplan-Meier methods were utilised to estimate the cumulative risk of recurrence up to five years after primary diagnosis, as well as the proportion of patients surviving free of recurrence. In patients who had evidence of recurrence, Kaplan-Meier methods were also used to estimate median survival time from the date of recurrence and the date of primary diagnosis. Cox proportional hazards models served to compare survival following recurrence by histological type, treatment, Breslow thickness and site of the primary invasion. A p-value of <0.05 was used in order to indicate statistical significance.

## Results

A total of 1063 patients and 1315 cSCCs were treated between January 1, 2007, and December 31, 2016. Of this, 118 patients did not meet the inclusion criteria; therefore, a total of 945 patients and 1197 cSCCs were included in the study with 70% of patients being male (n=662) and 30% being female (n=283). The mean age at primary cSCC presentation was 80 (range, 47-101) years. The mean follow-up period after diagnosis and treatment was 32 months. WLE was the treatment of choice for 79% of cSCCs (Table 1).

**Table 1:** Time trends of patients treated for cSCC with conventional WLE during the 10-year period cSCC: cutaneous squamous cell carcinoma;  WLE: wide local excision

Year	Number of cSCCs	WLE as treatment of choice, n (%)	p-value	Complete WLE, n (%)	p-value
Total	1197	945 (79)		873 (92)	
2007	29	29 (100)		27 (93)	
2008	55	52 (95)		46 (88)	
2009	69	67 (97)		65 (97)	
2010	114	104 (91)	<0.001	96 (92)	0.619
2011	107	81 (76)		72 (89)	
2012	133	106 (80)		101 (95)	
2013	167	121 (72)		112 (93)	
2014	150	110 (73)		102 (93)	
2015	187	152 (81)		141 (92)	
2016	186	123 (66)		111 (90)	

The scalp was the predominant site affected by cSCC (25%), followed by the ear and cheek at 19% and 16%, respectively (Table 2).

**Table 2:** Patient demographics, year, site of presentation, age, gender and treatment modality of recurrent cSCC and primary cSCC in patients without recurrence. WLE: wide local excision; C+C: curettage and cautery; cSCC: cutaneous squamous cell carcinoma

	Primary cSCC, n (%)	Recurrence, n (%)	p-value
Year			0.004
2016	186 (16)	2 (1.1)	
2015	187 (16)	0 (0)	
2014	150 (13)	3 (2.0)	
2013	167 (14)	4 (2.4)	
2012	133 (11)	6 (4.5)	
2011	107 (9)	1 (0.9)	
2010	114 (10)	5 (4.4)	
2009	69 (6)	6 (8.7)	
2008	55 (5)	1 (1.8)	
2007	29 (2)	1 (3.3)	
Location of cSCCs			<0.001
Scalp	310 (25)	19 (6.1)	
Ear	232 (19)	4 (1.7)	
Cheek	191 (16)	1 (0.5)	
Forehead	122 (10)	2 (1.6)	
Temple	130 (11)	3 (2.3)	
Nose	92 (8)	0	
Lips	37 (4)	0	
Upper Lip	19/37 (51)		
Lower Lip	18/37 (49)		
Neck	25 (2)	0	
Periorbital area	40 (3)	0	
Jaw Line	14 (1)	0	
Chin	4 (0.3)	0	
Age group (years)			0.514
≤ 69	141 (15)	3 (2.1)	
70-79	243 (26)	6 (2.4)	
80-89	424 (45)	13 (3.0)	
≥ 90	137 (14)	7 (4.5)	
Gender			0.022
Male	662 (70)	26 (3.8)	
Female	283 (30)	3 (1.1)	
Treatment modality			0.027
Mohs micrographic surgery	19	0	
WLE	945	18 (2)	
C+C	68	3 (6)	
Radiotherapy only	165	8 (5)	

Amongst all the patients, 191 (19.8%) developed a further primary cSCC in the head and neck area following an initial cSCC, of which 23% were synchronous (the two primaries were concurrent or within six months apart from each other) and 77% were metachronous (the second primary developed > six months after the first lesion).

Local recurrence was defined as tumour re-growth with comparable histology, with contiguity to the scar line which arises within the area (typically 2 cm) of the previously treated lesion. A regional recurrence is a tumour which spreads within the regional draining lymphatic basin [18]. 

**Figure 1:**
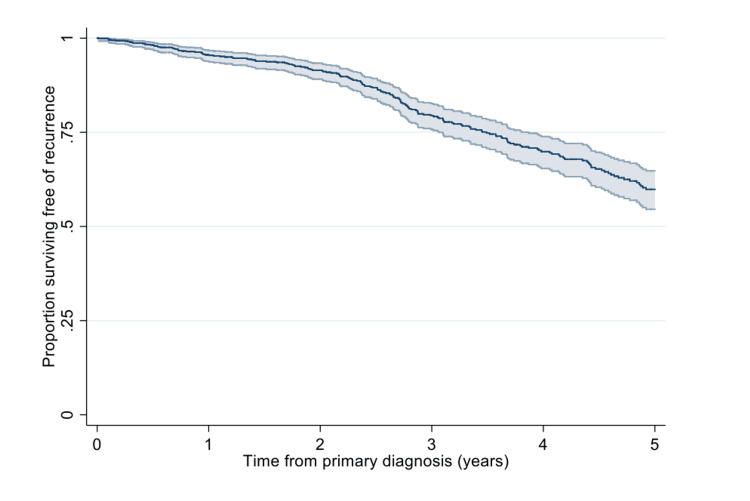
Kaplan-Meier estimate of the proportion of patients surviving free of recurrence.

In total, 29/945 (3%) patients had reoccurrences. The median time to recurrence was 25 (range, 1-81) months. Amongst the 29 recurrences, 26 were local, one regional, and two both local and regional. At one year after primary diagnosis, 95.6% (95%CI: 93.9%-96.8%) of all patients were recurrence-free compared to 59.9% (95%CI: 54.5%-64.8%) at five years (Figure 1).

**Figure 2:**
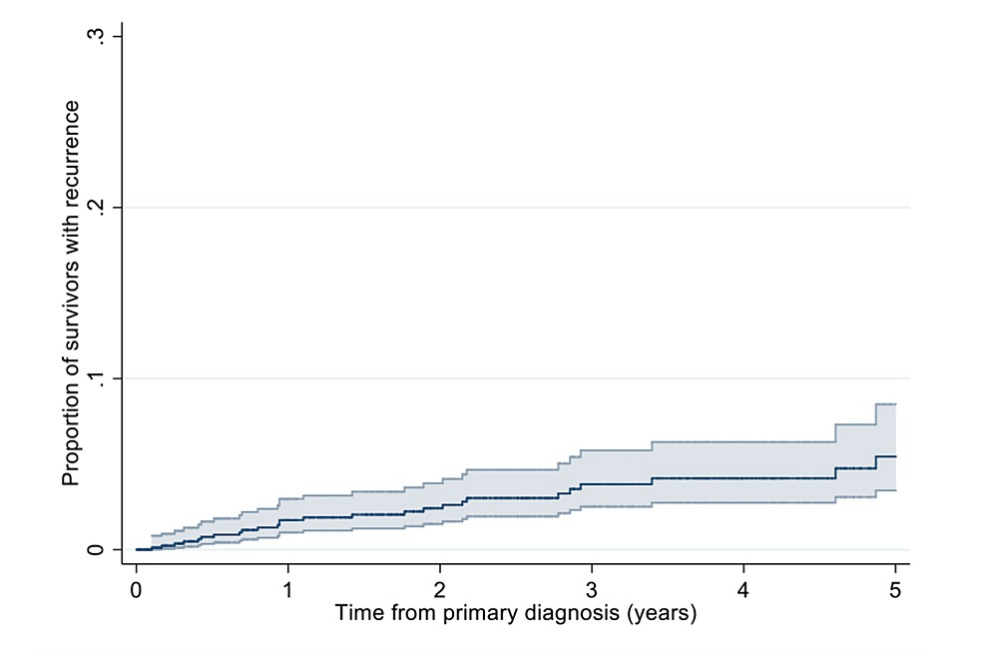
Kaplan-Meier estimate of the recurrence risk in survivors up to five years after primary cSCC diagnosis. cSCC: cutaneous squamous cell carcinoma

The risk of recurrence was 2.3% (95%CI: 1.1%-3.0%) one year after diagnosis. Of those still under follow-up at five years, 5.4% (95%CI: 3.5 to 8.5%) experienced a cSCC recurrence (Figure 2).

Recurrence rates (Table 2) varied by location of the cSSC (p<0.001), with the scalp being the most affected site. There were also significant variations by treatment type (p=0.027) with no recurrences observed with MMS following two years of follow-up. However, recurrence occurred within 6% of patients who were treated with C+C. The mean duration from the primary presentation to recurrence was 25 (range, 1-81) months.

**Table 3:** Tumour characteristics of recurrent cSCC and primary cSCC in patients without recurrence (variables such as perineural, intravascular and depth of invasion were calculated against the number of cSCCs treated with WLE and MMS). * Statistically significant; ** Bladder, prostate and lung cancer, chronic lymphocytic leukaemia, chronic myeloid leukaemia, multiple myeloma, and receiving corticosteroid treatment for chronic kidney disease, psoriasis, polymyalgia rheumatica, chronic obstructive pulmonary disease, and oral pemphigoid, type 1 and type 2 diabetes mellitus. Data re given as n (%) and mean (range) where indicated. cSCC: cutaneous squamous cell carcinoma;  WLE: wide local excision; MMS: Mohs micrographic surgery; H: head; N: neck; T2DM: type 2 diabetes mellitus

	Primary cSCC not causing recurrence, n (%)	Primary cSCC causing recurrence, n (%)	p-value
Total cSCCs treated	1168 (97.6)	29 (2.4)	
WLE + MMS	964 (82.5)	18 (62)	0.013*
Perineural invasion	47/964 (4.9)	8/18 (44)	<0.001*
Intravascular invasion	3/964 (0.3)	2/18 (11)	<0.001*
Depth of invasion			<0.001*
≤ 2 mm	529/964 (54.9)	2/18 (11)	
> 2 mm and ≤ 4 mm	296/964 (30.7)	5/18 (28)	
> 4 mm	132/964 (13.7)	11/18 (61)	
Histological type			<0.001*
Well differentiated	595/1168 (51)	4/29 (14)	
Moderately differentiated	437/1168 (37)	18/29 (62)	
Poorly differentiated	136/1168 (12)	7/29 (24)	
Positive lymphadenopathy		3/29 (10)	0.021*
Diameter > 20 mm	153/1168 (13)	18/29 (62)	<0.001*
Diameter primary cSCC, mean (range)		28 (8-130) mm	
Depth primary cSCC, mean (range)		5.6 (1-10) mm	
History of treatment			
Excision with +ve margin	68/964 (7)	5/18 (28)	0.008*
Only one cSCC	709/964 (73.5)	20/29 (69)	
≥ 2 cSCCs (H+N only)	191/964 (19.8)	9/29 (31)	
≥ 2 cSCC (H+N & body)	216/964 (22.4)		
Immuno-suppression**	102/1168 (8.7)	21/29 (72.4)	<0.001*
Patients with T2DM only		7/29 (24.1)	

Mean tumour diameter and mean depth of invasion in recurred tumours were respectively 28 (range, 8-130) mm and 5.6 (range, 1-10) mm. Perineural invasion was present in 5% of all lesions, increasing to 44% in the cohort with recurrence. Of the recurred cSCCs, 61% had a depth of invasion > 4 mm, whereas only 14% of all cSCCs infiltrated > 4 mm (p<0.001).

The majority of recurrent lesions were moderately (62%) and poorly (24%) differentiated with almost two-thirds (62%) having a diameter of > 20 mm (p<0.001). Out of 29 patients, 21 (72.4%) were immunosuppressed, of which one-third had type 2 diabetes mellitus (T2DM) (Table 3).

**Figure 3:**
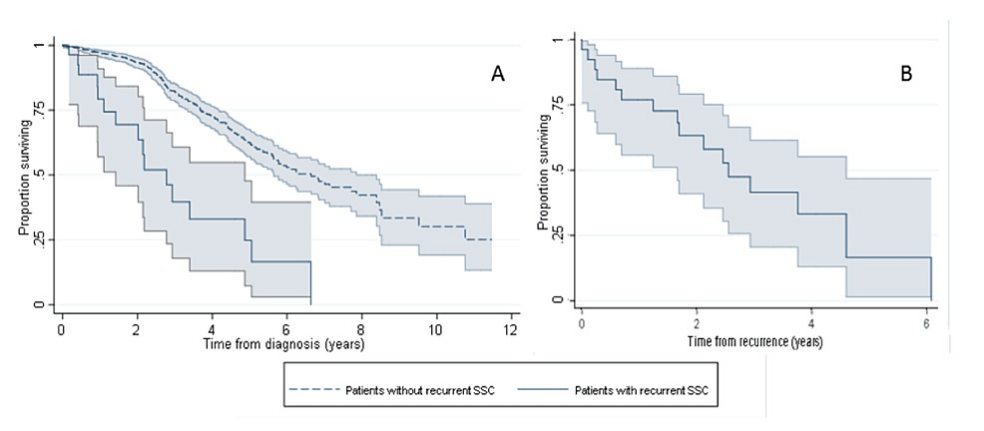
Kaplan-Meier estimate of (A) overall survival from diagnosis in patients who did and did not develop a recurrent cSCC and (B) survival following recurrent cSCC. cSCC: cutaneous squamous cell carcinoma

Of the 29 patients who had a reoccurrence, 16 died (55%). Median survival time from recurrence was 2.6 years (95%CI: 1.7-4.6) whereas from primary diagnosis it was 7.2 (95%CI: 3.9-8.2) years (Figure 3). There was no difference in survival following a recurrence in patients according to histological type (p=0.192), treatment (p=0.477), Breslow thickness (p=0.380), and site (p=0.245) of the primary invasion.

## Discussion

Our study highlighted several risk factors for the development of loco-regional recurrence, such as: (i) treatment modality, (ii) depth of invasion, (iii) diameter > 20 mm, (iv) gender, (v) histological differentiation, (vi) location of primary cSCC, (vii) perineural invasion, (viii) intravascular invasion, (ix) positive lymphadenopathy, (x) immuno-suppression and (xi) history of treatment for cSCC. Schmults et al. found that treatment modality and immunosuppression were not associated with an increased risk of recurrence [8].

Our results showed that radiotherapy led to a two-fold increase in reoccurrence in comparison to WLE (5% vs. 2%). In C+C, recurrence rates increased three-fold (6% vs. 2%). MMS was only performed in 19 cases (1.6%); nevertheless, there were no cases of recurrence after a two-year follow-up (p=0.027). Our study highlighted a loco-regional recurrence rate of 3%, which is in keeping with reported data [3,6,8].

Tumour diameter and depth of invasion, as highlighted in other studies [5,8,13], proved to be an important predictor for loco-regional recurrence. Where a depth of invasion of > 4 mm and a diameter of > 2 cm were present, the risk of developing a recurrence increased five-fold (61% vs. 14% and 62% vs. 13%, respectively). Rowe et al. reported a loco-regional recurrence rate of 30% for lesions that measured > 2 cm versus 9% for lesions that measured < 2 cm [12].

In our study, most patients with recurrent cSCCs were male (90%). It is believed that genetic, behavioural and occupational factors are important contributors to gender discrepancies [19].

Of recurrent cSCCs, 24% were poorly differentiated, in comparison with 12% in primary cSCCs reflecting histological differentiation as a risk factor for prognosis. These results corroborate previous studies on histological differentiation, with rates increasing up to four times (17% vs. 4%) [20]. A similar pattern was also highlighted in our study for moderately differentiated cSCCs, which accounted for the majority of the primary lesions leading to recurrences (62%) [2,12].

The most common sites for loco-regional recurrences were the scalp, ear and temple with 65%, 14% and 10%, respectively. In our study, only one recurrence (3%) developed on the cheek and no recurrences were reported on the lip. These data are in contrast with those of other authors who reported higher recurrence rates in the ear and lip with 18% and 13%, respectively [2,3,8,12]. However, considering the scalp is the most exposed region of the head and neck with the greatest surface area, it is reasonable to acknowledge why the scalp contributed to most of the primary and recurred primaries.

Perineural and intravascular invasions are uncommon, but they carry a high risk of recurrence [2,17]. Perineural invasion was found in 44% of the recurrent cSCCs and in 5% of the primary lesions treated successfully. 

Positive lymphadenopathy was another relevant risk factor for the development of loco-regional recurrences. It was found in three of the 29 patients who experienced recurrences of cSCC (10.3%). Other studies registered values as high as 30% [12,21].

Skin cancer in immunosuppressed patients tends to differ from that in healthy individuals. It occurs in patients who are 20-30 years younger than in age than those who are not immunocompromised, with an incidence 5-20 times higher than the normal population [22,23]. Our results are similar to those which have shown that immunosuppressed patients with cSCC more frequently exhibit high-risk pathological features [5,13-15]. A total of 72.4% of patients with recurrences were immunosuppressed; among them, 24.1% suffered from T2DM. Manyam et al. highlighted a loco-regional recurrence rate of 48% in their immunosuppressed population [14]. Foreman et al. analysed the relationship between T2DM and survival rates in patients with cSCC [24]. In their retrospective analysis of 319 patients, the authors concluded that T2DM alone does not adversely affect survival outcomes in head and neck cSCC.

Patients with incomplete excision (presence of positive margins) proved to be at more risk of developing loco-regional recurrences than those patients who did not require further treatment. Of incomplete excision, 28% belonged to the recurrence group compared to 7% of the non-recurrence group. Diagnosis of two or more primary cSCCs did not increase the risk of developing loco-regional recurrence (p>0.05).

Rowe et al. advocate that a long-term, possibly lifelong follow-up should be implemented for the early detection of recurrences [12]. In their systematic review and meta-analysis, they reported that 75% and 95% of loco-regional recurrences occurred within two and five years, respectively. The median time to reported recurrence in our study was 25 months (two years). After 2.5 years (67%) of follow-up, negative outcomes started to decrease, reaching a plateau after five years (90%). Only two recurrences occurred after the five-year period, at 5.5 and 6.7 years, respectively. The data suggests that long-term follow-up should be considered for all patients; however, it may be desirable to have this in specialist settings as the healthcare cost may be prohibitive.

This study has potential limitations. Reliance on data retrieval over a large period spanning 10 years provides certain challenges due to data being stored in various formats and details. The study also does not take into account occupational and environmental factors that may have an influence on reoccurrence and therefore does not capture the full complexity of our subjects. Although our research provides important insights into factors that may increase reoccurrence it does not incorporate the impact of other potentially influential variables such as surgical operator experience and type of closure following excision. Given the scope and resources of our study, we were confined to a single hospital within a specific city which has a certain demographic which may influence outcomes. 

## Conclusions

In light of the results of this study, the authors conclude that WLE and MMS provide superior recurrence-free curative outcomes than therapeutic C+C and radiotherapy as treatment of choice. History of incomplete excisions, diameter > 2 cm, positive lymphadenopathy, perineural invasion, and immunosuppression increase the risk of recurrence. Furthermore, gender is also a risk factor; however, it is highly dependent on modifiable and non-modifiable factors (the less likelihood of men using sun protection, occupations that require men to spend more time in the sun, and androgenic alopecia). The authors therefore do not consider gender as predictable as the other variables.

Moderately and poorly differentiated cSCCs are more likely to reoccur. The location of the lesion is significant and scalp and ear lesions recur more frequently than other sites in the head and neck region and the mean recurrence time of 25 months has led to our recommendation for implementing long-term follow-up reviews.
